# Depressive symptoms of older adults with chronic diseases: the mediating roles of activities of daily living and economic burden of diseases

**DOI:** 10.3389/fpsyg.2024.1387677

**Published:** 2024-07-02

**Authors:** Zihan Ni, Xiuyuan Zhu, Kan Tian, Qing Chen, Yi Yang, Shiyu Xie

**Affiliations:** ^1^School of Elderly Care Services and Management, Nanjing University of Chinese Medicine, Nanjing, China; ^2^School of Health Economics and Management, Nanjing University of Chinese Medicine, Nanjing, China; ^3^School of Public Health, Fudan University, Shanghai, China; ^4^National Health Commission Key Laboratory of Health Technology Assessment, Fudan University, Shanghai, China

**Keywords:** older adults, chronic diseases, activities of daily living, depressive symptoms, economic burden of diseases

## Abstract

**Objective:**

To explore the mediating roles of activities of daily living (ADL) and economic burden of diseases in the relationship between chronic diseases and depressive symptoms of older adults.

**Methods:**

The data were sourced from China Health and Retirement Longitudinal Study (CHARLS). The number of chronic diseases, ADL, out-of-pocket medical expenses and the Center for Epidemiological Studies Depression Scale (CES-D) were selected as measuring indexes. Mediation analysis was conducted to explore the potential mediating roles of ADL and economic burden of diseases in the association between chronic diseases and depressive symptoms.

**Results:**

The number of chronic diseases, ADL, economic burden of diseases and depressive symptoms of older adults were significantly correlated with each other. ADL and economic burden of diseases individually mediated the relationship between the number of chronic diseases and depressive symptoms, accounting for 31.460% and 5.471% of the total effect, respectively. Additionally, ADL and economic burden of diseases demonstrated a chain mediating effect in this relationship, contributing to 0.759% of the total effect.

**Conclusion:**

The chain-mediated model effectively elucidated the mediating roles of ADL and economic burden of diseases in the association between chronic diseases and depressive symptoms among older adults. The study underscores the need for policymakers to focus attentively on the mental health of older adults with chronic diseases. Enhancing the capacity for ADL and strengthening social security to mitigate the economic burden of diseases are recommended strategies to alleviate depressive symptoms in older adults.

## 1 Introduction

Population aging is anticipated to be one of the most significant social trends of the 21st century according to the United Nation’s Department of Economic and Social Affairs (2015). The increasing number and proportion of older individuals in all countries are set to have profound impacts on various aspects of economic and social development. As one of the most populous countries, China faces the formidable challenge of a rapidly aging population structure and an accelerated aging process (World Health Organization, 2019). Data from China’s seventh national population census revealed that individuals aged 60 and above constitute approximately 18.70% of the population, while those aged 65 and above made up 13.50% (National Bureau of Statistics of China, 2021). These figures mark China’s forthcoming transition toward a society characterized by moderate aging. Nowadays, improvements in the living environment, advancements in medical technology, strengthened disease prevention and control, and enhanced social welfare systems have extended the life expectancy of older adults (World Health Organization, 2019). However, the overall health status of this demographic remains a concern. Healthy life expectancy was issued by Sanders (1964). The concept emphasized the significance of keeping fit while extending life (Sun, 2023). However, while the average life expectancy in China reached 77 years in 2018, the healthy life expectancy was only 68.7 years (The National Health Commission of the People’s Republic of China, 2019). This discrepancy suggests that, on average, Chinese residents experience chronic diseases for over eight years. Relevant data indicated that approximately 180 million older individuals suffer from chronic diseases, with more than 75% diagnosed with one or more chronic diseases (Li et al., 2024). Chronic diseases not only have serious influences on their physical conditions (Sharma et al., 2021), but also adversely affect their mental health. Studies have shown that depression is one of the most common comorbidities among older individuals with chronic diseases (Sinnige et al., 2013). The prevalence of depression in patients with chronic diseases ranges from 9.3 to 25% (Ingle et al., 2017). In fact, individuals with multiple chronic diseases are twice as likely to suffer from depression compared to those without (Read et al., 2017). Moreover, the severity of depressive symptoms in older adults intensifies with the progression of chronic diseases (Birk et al., 2019), contributing to higher mortality rates (Cuijpers and Smit, 2002). Given the gradual decline in mental health among older adults, it is imperative to address the psychological challenges faced by this demographic (Batsis et al., 2021). Therefore, it is necessary to further investigate the relationship between chronic diseases and depressive symptoms in older adults to identify mechanisms that could mitigate these effects. In this mechanism, two potential mediators—activities of daily living (ADL) and economic burden of diseases—are considered. Chronic diseases often impair ADL and escalate the economic burden of diseases for older individuals, both recognized as significant predictors of depression (Meher et al., 2021; Mahumud et al., 2023). Thus, this study posits that ADL and economic burden of diseases may mediate the relationship between chronic diseases and depressive symptoms in older adults.

With the rise of age, older adults are more likely to experience declines in physiological function, deterioration of physical conditions and weakening of basic self-care abilities (Xu R. et al., 2019; Xu X. et al., 2019), together with an increased risk of chronic diseases and likelihood of complications. As a consequence, these factors may lead to the degeneration ADL (Su et al., 2016). In addition, different chronic diseases may differently impact activities of daily living. For instance, diabetic patients often suffer from multiple neuropathies, likely contributing to decreased muscle strength and thus impairing their ability to engage in physical activities (Hu et al., 2022). The decline in ADL can have detrimental effects on older adults. On the one hand, it impacts the autonomy of older adults, potentially fostering anxieties about the future and a sense of helplessness. Consequently, these factors may contribute to the onset of depression (Feng et al., 2021). Relevant studies indicated that the assessment of ADL serves as a more effective predictor of depression levels in older adults, heightened impairment in ADL correlates with increased severity of depression (Raj and Kanniammal, 2018). On the other hand, compromised ADL leads to a heightened necessity for medical attention and diagnostic assessments, thereby accruing medical expenses and elevating healthcare costs (Homma, 2016; Casanova and Lillini, 2021). This situation not only places a financial burden on older adults but also exacerbates the overall economic strain they face.

Furthermore, suffering from chronic diseases can amplify medical expenses and the incidence of catastrophic expenditures among older adults, diminishing their likelihood of continued labor participation and elevating their risk of poverty (Htet et al., 2014; Xu R. et al., 2019; Xu X. et al., 2019). Relevant studies indicated that individuals suffering from chronic diseases typically experience a higher frequency of medical visits and increased healthcare expenditures (Hong et al., 2022). Specifically, individuals afflicted with chronic heart disease are susceptible to substantial economic burdens (Mahumud et al., 2023). Patients with cerebrovascular disease, diabetes and chronic kidney disease are at significantly higher risk of catastrophic health expenditure (Geldsetzer et al., 2019). The financial stress caused by chronic diseases not only poses a challenge to the individuals, but also extends to their families. The significant financial strain can lead households to struggle with sustained elevated expenditures, potentially resulting in irregular treatment of chronic diseases and impoverishment (Counts and Skordis-Worrall, 2015). Additionally, this financial strain might compel older adults to cut back on expenditures in other essential areas, impacting their ability to meet basic needs and exacerbating their overall situation, thereby increasing their vulnerability to depressive symptoms (Yi and Syrjala, 2017).

Although existing studies have primarily focused on exploring the relationships between chronic diseases and depressive symptoms among older adults (Park et al., 2018; Guo et al., 2023). Few researchers have considered the potential impact of chronic diseases on the degeneration in ADL and the resulting economic burden of diseases on the older adults, which may contribute to depressive symptoms. Exploring these mechanisms is relevant not only to the personal physical and mental well-being as well as quality of life of older adults but also to the improvement of the social healthcare system and related policies. Clarifying the relationships between these variables can provide a fresh research perspective and pathways for health improvement from physiological, psychological, and economic aspects. Therefore, this study endeavors to investigate an influential mechanism through which the decline in ADL and the rise of economic burden of diseases contribute to depressive symptoms in the older individuals with chronic diseases. It is helpful to enhance comprehensive understanding of chronic diseases in older adults through empirical analysis and to offer valuable possibilities for the formulation of intervention measures, enhancement of quality of life, and promotion of overall well-being among older adults.

## 2 Materials and methods

### 2.1 Sample selection

The study utilized the data from the fourth phase (2018) of the China Health and Retirement Longitudinal Survey (CHARLS), conducted by Peking University. CHARLS aims to collect a set of high-quality micro-data representative of households and individuals aged 45 and above, encompassing 150 country-level units and 450 village-level units across China. The CHARLS comprises fundamental information pertaining to individual demographics, household characteristics, health status and functioning, working and retirement, pensions and various other aspects. It is designed for large-scale national research, allowing for the measurement of both physical conditions and mental health. The CHARLS National Baseline Survey was launched in 2011 and was tracked 2–3 years. In order to guarantee the unbiased and representativeness of the survey, CHARLS utilized two methods. Firstly, it set a filtering section that can exclude invalid samples. Secondly, CHARLS conducted the sample through four phases at the county (district)-village (residential)-household-individual level (Tang et al., 2022a,b). CHARLS used the Probability Proportional to Size (PPS) method for sampling at two levels: county (districts)-village (residential). Initially, 150 counties and districts were randomly selected, from which 3 villages/communities were randomly chosen, obtaining a total of 450 villages/communities. CHARLS utilized its specially designed mapping software (CHARLS-GIS) for on-site mapping and household information collection in each village or community. They randomly selected 25–36 households from the map to determine the sample size for each household.

**Figure 1:**
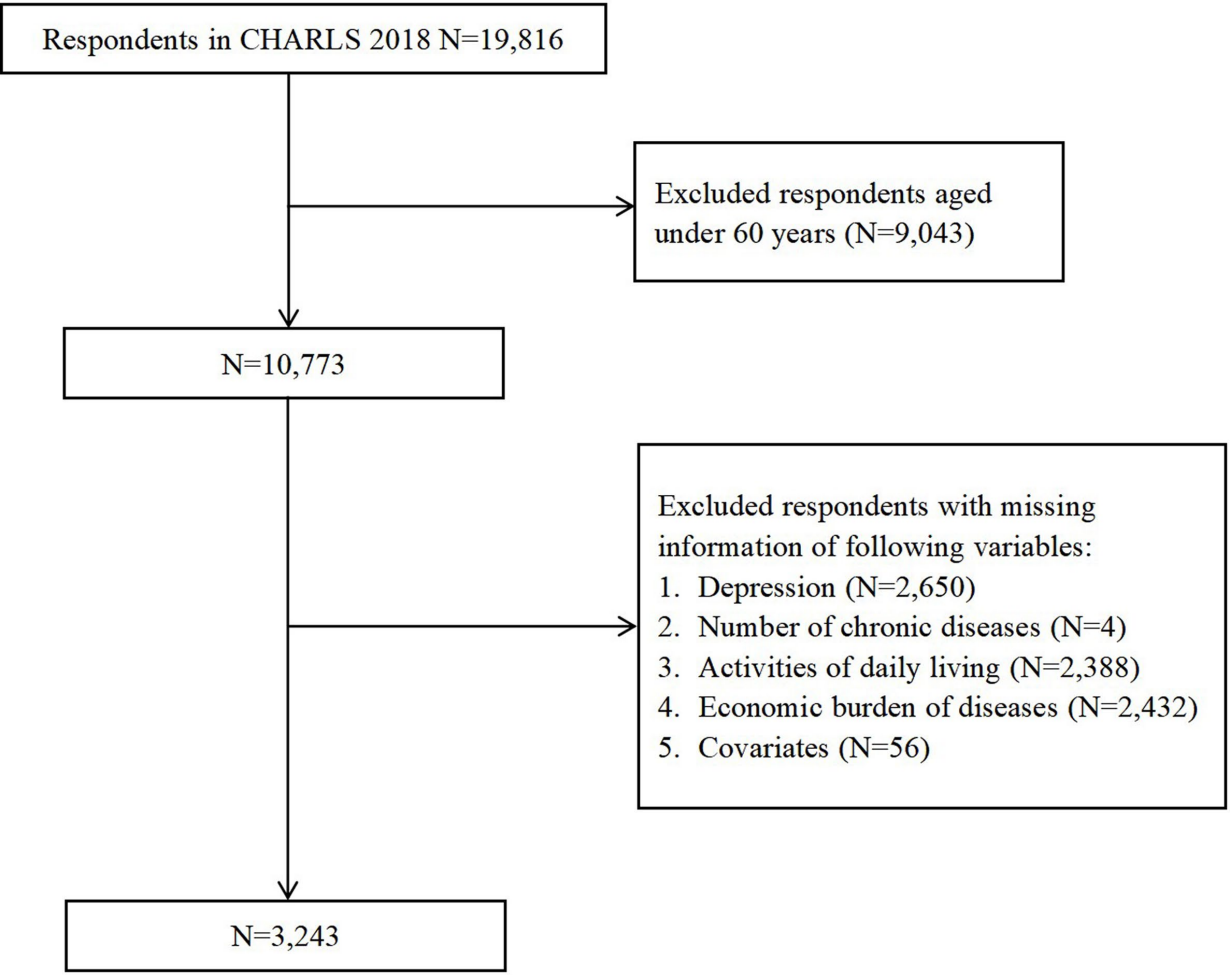
Respondents’ flow in the study.

By the end of the 2018 follow-up, the sample comprised a total of 19,816 respondents across 12,400 households, establishing a relatively high-quality data set. Since the research object is the older adults in this study, 9,043 respondents under the age of 60 were excluded. Among the remaining respondent, 2,650 were missing information on depressive symptoms, 4 were missing information on chronic diseases, 2,388 were missing information for ADL, 2,432 were missing information for economic burden of diseases and 56 were missing information for covariates. 3,243 respondents were finally included in our research and the sample selection process is depicted in Figure 1.

### 2.2 Measures

#### 2.2.1 Dependent variable

Depressive symptoms was the dependent variable in this study. The presence of depressive symptoms of older adults with chronic diseases were measured by the Center for Epidemiological Studies Depression Scale (CES-D) provided in the questionnaire. The scale comprises 10 items and can be divided into 2 parts. For the negative item, the scale contains 8 questions, including whether the older adults are bothered by small things, having difficulty concentrating, feeling depressed, struggling to do things, feeling fearful, sleeping are restless, feeling lonely, could not get “going”. The positive item covers 2 questions, including whether the older adults are feeling hopeful about the future and feeling happy. The ten questions of the scale were scored on a four-scale metric, ranging from “rarely or none of the time” to “some or a little of the time”, “occasionally or a moderate amount of the time” and “most or all of the time”. Scores ranged from 0 for “rarely or none of the time” to 3 for “most or all of the time” for negative questions, while the two positive questions were reverse scored (Lei et al., 2014). The scores of the ten questions were added together to construct a comprehensive index of depressive symptoms in older adults, with the value ranging from 0 to 30. The depressive symptoms score was a continuous variable. According to the detection rate standard commonly used (Santor and Coyne, 1997), scores below 10 were categorized as indicative of an absence of depressive symptoms, whereas scores of 10 or higher suggest the presence of depressive symptoms. Higher scores indicated more severe depressive symptoms in older adults. The internal heterogeneity coefficient (Cronbach’s α) of the depression scale employed in this study was 0.814, greater than 0.7, indicating a high level reliability of data and robust reliability of measurement.

#### 2.2.2 Independent variable

The independent variable of this study was number of chronic diseases of older adults. CHARLS includes a total of 14 chronic diseases. The study measured the presence of chronic diseases among older adults based on doctor diagnoses. The specific questions include the following: (1) Have you been diagnosed with Hypertension by a doctor? (2) Have you been diagnosed with Dyslipidemia (elevation of low density lipoprotein, triglycerides (TGs), and total cholesterol, or a low high density lipoprotein level) by a doctor? (3) Have you been diagnosed with Diabetes or high blood sugar by a doctor? (4) Have you been diagnosed with Cancer or malignant tumor (excluding minor skin cancers) by a doctor? (5) Have you been diagnosed with Cancer or malignant tumor (excluding minor skin cancers) by a doctor? (6) Have you been diagnosed with Liver disease (except fatty liver, tumors, and cancer) by a doctor? (7) Have you been diagnosed with Heart attack, coronary heart disease, angina, congestive heart failure, or other heart problems by a doctor? (8) Have you been diagnosed with Stroke by a doctor? (9) Have you been diagnosed with Kidney disease (except for tumor or cancer) by a doctor? (10) Have you been diagnosed with Stomach or other digestive diseases (except for tumor or cancer) by a doctor? (11) Have you been diagnosed with Emotional, nervous, or psychiatric problems by a doctor? (12) Have you been diagnosed with Memory-related disease (such as dementia, brain atrophy, and Parkinson’s disease) by a doctor? (13) Have you been diagnosed with Arthritis or rheumatism by a doctor? (14) Have you been diagnosed with Asthma by a doctor? However, the dependent variable in this study was depressive symptoms of older adults, and having emotional, nervous, or psychiatric problems may be highly correlated with depressive symptoms. Consequently, the study excluded emotional, nervous as well as psychiatric problems to eliminate potential confounding effects. Ultimately, the study encompassed 13 categories of chronic diseases. If the answer was yes, a value of 1 was assigned; if the answer was no, a value of 0 was assigned. The final count of chronic diseases among older adults was obtained by summing up these values based on the participants’ responses.

#### 2.2.3 Mediators

The mediating variables in this study included activities of daily living (ADL) and the economic burden of diseases in older adults with chronic diseases. In the current literature, both activities of daily living (ADL) and instrumental activities of daily living (IADL) are widely recognized as comprehensive assessments of the independent living ability of older adults (Chauhan et al., 2022). In the CHARLS, the measuring items for ADL scale include: difficulty with dressing, difficulty with bathing or showering, difficulty with eating, difficulty with getting into or out of bed, difficulty with using the toilet, difficulty with controlling urination and defecation. The measuring items for IADL scale include: difficulty with doing household chores, difficulty with preparing hot meals, difficulty with shopping for groceries, difficulty with making phone calls, difficulty with taking medications, difficulty with managing money. A 4-point scoring system was employed for all answers in this scale and the four response options were 0 = “No, I do not have any difficulty”, 1 = “I have difficulty but can still do it”, 2 = “Yes, I have difficulty and need help”, 3 = “I cannot do it”. To standardize the scoring index, the question “Because of health and memory problems, do you have any difficulties with making phone calls?” was excluded due to the challenge of measuring this index for individuals without a phone at home. Subsequently, scores from the remaining 11 questions were summed to construct a comprehensive ADL index. A higher score on this index indicated greater impairment.

As the CHARLS data set did not include the indirect economic burden of diseases, our study focused solely on the direct economic burden. This encompassed expenses related to outpatient care, self-care, inpatient care, along with non-medical expenses. Specifically, the economic burden of outpatient care was calculated as the sum of outpatient care and transportation expenses in the month preceding the interview multiplied by 12. The economic burden of self-care was determined by the self-medical expenditure in the month before the interview multiplied by 12. For inpatient care, the economic burden included fees paid to the hospital, transportation expenses, nutrition expenses, family expenses and accommodation expenses in the year preceding the interview, following the methodology outlined in previous studies (Shi et al., 2017; Guo et al., 2021). To facilitate statistical analysis, logarithmic transformation was applied to the economic burden of diseases.

#### 2.2.4 Covariates

The control variables selected for this study encompassed gender (0 = female, 1 = male), age (in years), education level (0 = low level of education, including elementary school and below; 1 = medium level of education, including middle school graduation to vocational school; 2 = high level of education, including Two-/Three-Year College/Associate degree and above), marital status (0 = married, 1 = separated/divorced/widowed/never married), sleep duration(in hours), smoking (0 = non-smoking, 1 = smoking), drinking (0 = non-drinking, 1 = drinking but less than once a month, 2 = drinking more than once a month), social participation (0 = no social participation, 1 = social participation) and health insurance participation (0 = no health insurance participation, 1 = health insurance participation).

### 2.3 Statistical analysis

A descriptive analysis was conducted to characterize participant profiles, employing percentages for discrete variables and means with standard deviations for continuous variables. Subsequently, multiple linear regression was employed to investigate the effects of the independent variable and mediating variables on the dependent variable after controlling covariates. Before examining mediating effects, the research conducted Harman’s single-factor test on all entries to identify potential common method bias. Additionally, partial correlation analysis was performed to mitigate the influence of mediating variables on other potential factors, ensuring a more accurate assessment of the mediating effect. The study utilized the bias-corrected percentile Bootstrap method with the SPSS macro developed by Hayes (Model 6). Controlling for gender, age, education level, marital status, sleep duration, smoking, drinking, social participation and health insurance participation, 5,000 samples were extracted to estimate the 95% confidence interval for the mediation effect. A mediation effect was considered significant if the 95% confidence interval did not include zero (Preacher et al., 2007). All statistical analyses were conducted using IBM SPSS Statistics version 27.

## 3 Results

### 3.1 Descriptive statistics

The descriptive results of the data were presented in Table 1. The sample comprised 3,243 individuals, with 47.209% male and 52.791% female. Among the participants, 76.0% had a low level of education, while 22.510% had a medium education level, respectively. Approximately 70% of older participants were married. The average age was 69.170 years, with a standard deviation of 6.457. The mean number of chronic diseases was 3.207, indicating a significant level of multimorbidity among the older adults. The mean depressive symptoms score in the sample was 10.510, with a standard deviation of 6.952. Based on the commonly used detection rate standard (Santor and Coyne, 1997), 49.399% of the older adults with chronic diseases exhibited depressive symptoms, underscoring a considerable prevalence of depressive symptoms within the sample.

**Table 1:** Descriptive statistics of variables (*n* = 3,243).

Variables		Mean/%	SD	Minimum	Maximum
Dependent variable	Depressive symptoms	10.510	6.952	0	30
Independent variable	Number of chronic diseases	3.207	1.888	1	13
Mediators	Activities of daily living	2.258	3.957	0	32
	Economic burden of diseases	8.390	1.491	2.303	14.004
Covariates	Demographic factors				
	Gender				
	male	47.209%			
	female	52.791%			
	Age	69.170	6.457	60	95
	Education level				
	Low level of education	76.010%			
	Medium level of education	22.510%			
	High level of education	1.480%			
	Marital status				
	Separated/divorced/widowed/never married	30.003%			
	married	69.997%			
	Health factors				
	Number of chronic diseases	3.207	1.888	1	13
	Activities of daily living	2.258	3.957	0	32
	Sleep duration	5.864	2.111	0	15
	Smoking			0	1
	Non-smoking	72.957%			
	Smoking	27.043%			
	Drinking	0.485	0.815	0	2
	Social factors				
	Social participation	0.500	0.500	0	1
	Health insurance participation	0.972	0.164	0	1

### 3.2 Multiple linear regression

The study employed multiple linear regression to construct two models, investigating the effects of the number of chronic diseases, ADL and economic burden of diseases on depressive symptoms after controlling for covariates. Statistical multicollinearity problems tend to occur when tolerance is less than 0.2 or 0.1 and variance inflation factors (VIF) are greater than 5 or 10. The tolerance of predictors ranged from 0.631 to 0.985, and VIFs ranged from 1.015 to 1.585 in this study, indicating no significant multicollinearity issues. The value of the Durbin-Watson statistic was 1.866, which suggested the absence of autocorrelation. The results of the multiple linear regression were detailed in Table 2. In model 1, only covariates were included in the regression equation and the adjusted *R*^2^ was 0.123. In model 2, the number of chronic diseases, ADL and economic burden of diseases were added on the basis of covariates. The adjusted *R*^2^ was 0.211, reflecting an 8.8% increase in explained variance. Number of chronic diseases (*β* = 0.410, *p* < 0.01), ADL (*β* = 0.434, *p* < 0.01) and economic burden of diseases (*β* = 0.198, *p* < 0.05) positively predicted depressive symptoms in the older adults.

**Table 2:** Multiple linear regression of Depressive symptoms of older adults with chronic diseases.

		Model 1	Model 2
Covariates	Demographic factors		
	Gender		
	Male (reference group: female)	−1.267***	−1.289***
	Age	−0.074***	−0.109***
	Education level (Reference group: low level of education)		
	Medium level of education	−1.947***	−1.735***
	High level of education	−4.070***	−3.578***
	Marital status		
	Separated/divorced/widowed/never married (reference group: married)	1.116***	1.002***
	Health factors		
	Sleep duration	−0.763***	−0.611***
	Smoking		
	Smoking (reference group: non-smoking)	0.881***	0.997***
	Drinking	−0.579***	−0.334**
	Social factors		
	Social participation (reference group: no social participation)	−1.260***	−0.977***
	Health insurance participation (Reference group: no health insurance participation)	−0.168	0.159
Independent variable	Number of chronic diseases		0.410***
Mediators	ADL		0.434***
	Economic burden of diseases		0.198**
Adjustment *R*^2^		0.123	0.211
*F*-value		46.648***	67.548***

*, **, and *** indicate significant at the 10, 5, and 1% levels, respectively.

### 3.3 Harman’s single-factor test

Lack of consideration of common method effects in empirical research can result in several negative consequences for the interpretation of outcomes, such as bias in the estimates of the relationships between constructs of interest. Therefore, before exploring the mediating role of ADL and the economic burden of diseases in the relationship between chronic diseases and depressive symptoms among older adults, the study conducted Harman’s single-factor test. This was completed to check for potential common method bias, ensuring that the results of the mediation analysis would be more accurate and reliable. Ultimately, the test extracted four common factors with eigenvalues greater than 1. The percentage of variance explained for the first common factor was 17.072%, well below the critical criteria of 40%, indicating negligible common method bias.

### 3.4 The partial correlation analysis

The analysis results presented in Table 3 demonstrate statistically significant positive correlations among the variables of the number of chronic diseases, ADL, economic burden of diseases, and depressive symptoms. These correlations established the foundation for further the mediating effect analysis.

**Table 3:** Results of partial correlation analysis.

Variable	*M* (SD)	1	2	3	4
1 Number of chronic diseases	3.207 (1.888)	1			
2 Activities of daily living	2.258 (3.957)	0.230***	1		
3 Economic burden of diseases	8.390 (1.491)	0.255***	0.182***	1	
4 Depressive symptoms	10.510 (6.952)	0.186***	0.289***	0.120***	1

*, **, and *** indicate significant at the 10, 5, and 1% levels, respectively.

### 3.5 Mediating effect test

**Figure 2:**
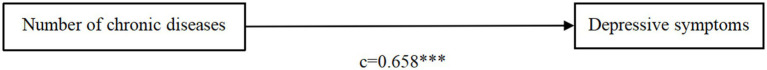
Direct effect path diagram. ***p < 0.01.

**Figure 3:**
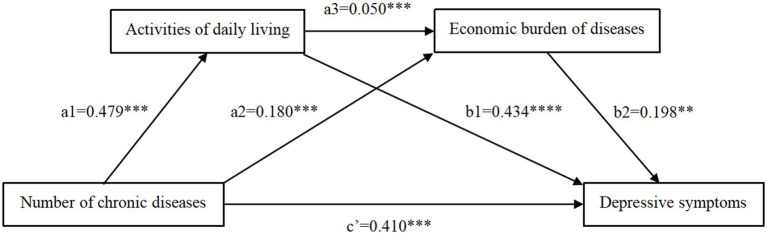
Chain mediation effect path diagram. ***p < 0.01, **p < 0.05.

The study further investigated the mediating roles of ADL and the economic burden of diseases between number of chronic diseases and depressive symptoms. The results were illustrated in Table 4, as well as Figures 2, 3. Figure 2 revealed that number of chronic diseases positively predicted ADL (path a1: *β* = 0.479, *p* < 0.01). Table 4 and Figure 3 indicated three models. First, number of chronic diseases positively correlated with depressive symptoms (path c: *β* = 0.658, *p* < 0.01). Second, number of chronic diseases (path a2: *β* = 0.180, *p* < 0.01) and ADL (path a3: *β* = 0.050, *p* < 0.01) were positive predictors of economic burden of diseases. Third, both ADL (path b1: *β* = 0.434, *p* < 0.01) and economic burden of diseases (path b2: *β* = 0.198, *p* < 0.05) significantly predicted depressive symptoms.

**Table 4:** Regression analysis of the relationship of variables.

Model		Fit index			Significance	
Outcome variable	Predictor variable	*R*	*R* ^2^	*F*	β	*t*
Activities of daily living	Gender	0.350	0.123	45.146***	−0.197	−1.198
	Age				0.065	6.031***
	Education level				−0.699	−4.869***
	Marital status				0.527	3.404***
	Social participation				−0.919	−6.919***
	Health insurance participation				−1.109	−2.775***
	Sleep duration				−0.163	−5.144***
	Smoking				0.206	1.222
	Drinking				−0.261	−2.985***
	Number of chronic diseases				0.479	13.447***
Economic burden of diseases	Gender	0.332	0.111	36.494***	−0.057	−0.922
	Age				0.004	1.046
	Education level				0.257	4.707***
	Marital status				−0.423	−7.189***
	Social participation				0.035	0.692
	Health insurance participation				0.245	1.611
	Sleep duration				−0.013	−1.042
	Smoking				−0.124	−1.936*
	Drinking				−0.093	−2.799***
	Number of chronic diseases				0.180	12.977***
	Activities of daily living				0.050	7.489***
Depressive symptoms	Gender	0.463	0.214	73.198***	−1.288	−4.716***
	Age				−0.109	−6.101***
	Education level				−1.748	−7.274***
	Marital status				1.003	3.858***
	Social participation				−0.977	−4.391***
	Health insurance participation				0.159	0.238
	Sleep duration				−0.611	−11.568***
	Smoking				0.997	3.554***
	Drinking				−0.334	−2.294**
	Number of chronic diseases				0.410	6.575***
	Activities of daily living				0.434	14.701***
	Economic burden of diseases				0.198	2.566**

*, **, and *** indicate significant at the 10, 5, and 1% levels, respectively.

The non-parametric bootstrap test in Table 5 and Figure 3 confirmed significant mediating effect, with a total indirect effect of 0.248. Three mediating pathways were identified. ADL and economic burden of diseases significantly mediated the effects of number of chronic diseases on depressive symptoms (indirect effect 1: 0.208, 31.611% of the total effect; indirect effect 2: 0.036, 5.471% of the total effect). Additionally, ADL and economic burden of diseases played a chain-mediated role in the relationship between number of chronic diseases and depressive symptoms (indirect effect 3: 0.005, 0.759% of the total effect). The 95% confidence intervals for these indirect effects did not include zero, validating the significance of these mediating paths.

**Table 5:** Analysis of intermediary effects.

	Indirect effect value	Boot SE	Boot LL CI	Boot UL CI	Relative mediation effect
Total effect	0.658	0.061	0.538	0.779	
Direct effect	0.410	0.062	0.288	0.533	62.310%
Total indirect effect	0.248	0.028	0.194	0.304	37.690%
Indirect effect 1	0.208	0.023	0.164	0.255	31.460%
Indirect effect 2	0.036	0.015	0.008	0.065	5.471%
Indirect effect 3	0.005	0.002	0.001	0.009	0.759%

Indirect effect 1, number of chronic diseases → activities of daily living → depressive symptoms; indirect effect 2, number of chronic diseases → economic burden of diseases → depressive symptoms; indirect effect 3, number of chronic diseases → activities of daily living → economic burden of diseases → depressive symptoms.

## 4 Discussion

The research examined the impact of the number of chronic diseases on depressive symptoms in the older adults, as well as the chained mediating roles of ADL and the economic burden of diseases in this relationship. The study identified four pathways linking chronic diseases to depressive symptoms, including one direct and three indirect routes. Directly, the prevalence of chronic conditions correlates with depressive symptoms. This is attributed to the persistent nature of such diseases, characterized by high incidence, lengthy duration, uncertain outcomes, and lack of complete curability. Consequently, patients endure persistent symptoms, such as pain, fatigue, and insomnia, over an extended period (Li et al., 2023). Furthermore, most patients need to rely on medication for extended periods, and the cumulative effects of medication side effects eventually lead to depression and other adverse emotions through endocrine mechanisms (Kim et al., 2023). Moreover, cognitive decline or memory loss in older adults can pose challenges in adhering to medication regimens, potentially diminishing the treatment efficacy and exacerbating depressive symptoms.

Indirectly, one pathway illustrates how chronic illnesses among older adults impact depressive symptoms through diminished ADL capabilities. On the one hand, chronic diseases may impair the ability of older adults to perform daily tasks, resulting in their inability to perform some basic daily tasks independently (Chen et al., 2018). Older adults experiencing ADL disorders might require sustained assistance from others, fostering feelings of dependency and self-perception as a burden to family, which heightens susceptibility to depression (Yuan et al., 2022). On the other hand, due to physical discomfort and decreased mobility, older adults may decrease social interactions (Loke et al., 2015), including participation in social events, outdoor activities, and family gatherings. This decline can contribute to heightened feelings of loneliness and isolation among older adults, subsequently elevating their risk of experiencing depression.

Another indirect pathway involves the influence of chronic diseases on depressive symptoms in older adults, mediated by the economic burden of diseases. A plausible explanation is that chronic diseases typically have a protracted course and present challenges in terms of recovery. Older adults with chronic diseases require frequent medical treatment and the accumulation of medical costs may exceed the economic affordability, generating financial stress and anxiety, which may precipitate depressive symptoms (Brinda et al., 2012). In addition, people with chronic diseases often require long-term medication to maintain their health. However, older adults have limited pensions or retirement funds, the concerns over depleting savings or burdening family members due to ongoing medical expenses can further contribute to stress and depression (Choi et al., 2015).

Moreover, number of chronic diseases in older adults further exacerbate depressive symptoms through the chain-mediated effect between ADL and the economic burden of diseases. In this relationship ADL serve as a mediator in the association between the number of chronic diseases and the economic burden of diseases. This is primarily because the physical impairments and dysfunctions resulted from chronic diseases can negatively impact an individual’s ability to perform activities of daily living (Kim, 2011), and the coexistence of multiple chronic diseases has been proved to be strongly associated with disability (Chen and Sloan, 2015). In turn, diminished activities of daily living not only contributes to increased dependence on medical and nursing services, but may also prolong the rehabilitation and treatment process, escalating the demand for healthcare resources and financial expenditures (Prado and Jamora, 2020). Specifically, patients with chronic diseases reduced mobility due to chronic diseases may require more frequent medical examinations, long-term rehabilitation plans, more extensive professional nursing services, and even the use of special assistive devices. The provision of these services incurs substantial costs (Langa et al., 2002). As a result, older people and their families often face long-term and costly treatment costs. This can significantly impact their financial stability and quality of life, further contributing to the impoverishment of older adults. When the risk of falling into economic poverty increases, older people with chronic diseases experience heightened levels of depression (Della Corte et al., 2020).

Building upon the aforementioned points, this paper advocates for a multifaceted approach to alleviate depressive symptoms in the older adults with chronic conditions. Firstly, pay more attention to the mental health status of older individuals with chronic diseases. The study indicated that depressive symptoms is more pronounced in Chinese older individuals suffering from chronic diseases, which can also further lower the living standards and affect the quality of life of older adults, so it is urgent to take measures to alleviate this phenomenon. Community health service centers should attach importance to the mental health of older adults with chronic diseases (Wang et al., 2018), provide regular psychological examination and counseling services, and carry out mental health education activities for older adults with chronic diseases. It aims to heighten their awareness of their physical conditions, bolster their confidence in recovery, and reinforce their overall zest for life, thereby inspiring them to adopt a more positive outlook.

Secondly, enhance the ability of daily living of older adults with chronic diseases. The ability of daily living is closely related to the quality of life and health satisfaction of older individuals. Improving the physical ability of older adults can effectively prevent the development of physical diseases and positively impact their mental health, thereby reducing related health expenditures (Parra-Rizo, 2018; Agustí et al., 2023). Comprehensive health management of older patients with chronic diseases should be strengthened (Kim and Ha, 2020), and relevant policymakers should take measures to enhance the service capacity of primary medical and health institutions. The related actions include offering health consultation services, establishing health archives for older adults and encouraging them to participate in regular and comprehensive checkups, such as screening and assessment of chronic diseases and related health risks. It is also crucial to further expand the coverage of family doctor services. Family doctors play a key role in the management of chronic diseases among older adults (Huang et al., 2022). They often possess a more comprehensive understanding of their patients’ physical and mental conditions, enabling them to develop personalized rehabilitation and health management plans that are better tailored to each patient’s needs. The government can stimulate the capacity of family doctors by increasing financial support for them, expanding the scope of services and setting up more family doctor service stations. Furthermore, the application of Chinese medicine technology plays a significant role in preventing and treating the decline of physiological functions and enhancing the life activities of older adults with chronic diseases (Sun et al., 2017). Currently, health management and disease treatment efforts are increasingly focused on the rehabilitation of older adults with diseases. Another vital aspect is “preventive treatment” which emphasizes reinforcing physical conditioning in older adults. This approach is designed to help them avoid illnesses, improve their overall fitness, and strengthen their health, ultimately preventing the onset or worsening of chronic conditions that could lead to incapacitation. By promoting the widespread adoption of specialized diagnostic and therapeutic technologies in Chinese medicine—including traditional herbs, acupuncture, massage, and cupping—it is possible to enhance the effectiveness of both preventive and curative measures (Tang et al., 2022a,b). This approach aims to further bolster the physical and mental well-being of older adults. Additionally, accelerating the construction of livable communities is also an effective strategy to enhance activities of daily living of older adults with chronic diseases (Black and Jester, 2020). Government authorities and policymakers should intensify efforts to tailor living environments to meet the needs of the aging population. This involves creating health-supportive environments and enriching cultural elements to develop living spaces that foster the well-being of older adults. These measures aim to simplify the lives of older individuals and reduce the prevalence of depressive symptoms among them.

Thirdly, enhancing financial protection for older individuals managing chronic diseases and disabilities is crucial. Government authorities and relevant policymakers should improve and streamline the disability assessment process, providing suitable subsidies to older adults based on assessment outcomes. Specifically, there is a pressing need to strengthen financial assistance for older adults facing substantial levels of disability. In addition, expanding serves as an effective strategy to mitigate the catastrophic medical costs associated with disabilities (Hu et al., 2020). Long-term care insurance can not only alleviate the financial burden of older adults, but also enhance their physical health and provide emotional support, thereby reducing their depressive symptoms.

## 5 Strengths and limitations

This study explored the chain-mediated effect of activities of daily living and economic burden of diseases in the relationship between chronic diseases and depressive symptoms in older adults. While existing research mainly focused on the association between chronic diseases, activities of daily living, and depressive symptoms among older adults, this research innovatively introduced the economic burden of diseases as a mediating variable. It explored how the number of chronic diseases increases financial burden and influences depressive symptoms, providing a fresh perspective on the impact of chronic diseases on depressive symptoms in older adults. Through underscoring the critical role of the economic burden of diseases in this intricate chain of relationships, this study unveiled the dual impact of chronic diseases on both the quality of life and mental health of older individuals. The insights are crucial for developing comprehensive policies that address chronic disease management and provide mental health support for older adults. The main social significance of this study lies in advocating for attention from various sectors of society to the physical and mental health conditions of older individuals suffering from chronic illnesses. It also aims to address the personal, familial, and socioeconomic burdens that arise as a result of these conditions.

However, the study has several limitations. Initially, it used the number of chronic diseases as the independent variable, without categorizing the types of chronic diseases. Furthermore, it did not delve into the potentially different impacts of single versus multiple chronic diseases (comorbidities) on depressive symptoms in the older adults, which warrants further investigation. Secondly, China piloted long-term prescription policy in some regions in 2015 and officially introduced a national policy in 2021, which stipulates that patients with chronic disease can be prescribed up to 3 months of medication at a time. Although this study utilized data from CHARLS 2018, when pilot areas were limited, this could still introduce a bias in the calculation of economic burden of diseases. We will refine and calculate economic burden of diseases based on regional policies in the future research. Additionally, CHARLS lacks indicators for measuring the indirect economic burden of diseases, which could prevent a full understanding of the economic challenges faced by older adults in managing chronic diseases. Therefore, future studies may consider introducing more detailed measurement tools to examine the impact on family dynamics, social interactions, and productivity. This approach aims to comprehensively and accurately assess the multifaceted impact of chronic illnesses on the economic burden experienced by older adults.

## 6 Conclusion

With the widespread promotion on concepts of active aging and healthy aging, there is growing global focus on the mental health of older adults. Chronic diseases are significant risk factors that exacerbate depressive symptoms in the older adults, need to be closely monitored. This study primarily explored the impact of the number of chronic diseases on depressive symptoms among the older adults and the potential mechanisms involved. It illustrated multiple pathways between the number of chronic diseases and depressive symptoms in older adults. The direct pathway suggested that the number of chronic diseases can directly influence level of depressive symptoms, which may be related to the negative impacts of long-term symptoms and medication. Indirect pathways encompassed the influence of chronic diseases on depressive symptoms in older adults through several mechanisms: by affecting their ability to carry out daily activities, thereby exacerbating depressive symptoms; by increasing the financial burden of diseases; through impairing their ability to perform activities of daily living, leading to an elevated financial burden of diseases. Therefore, this study proposes that in the future we can alleviate the depressive symptoms of older adults with chronic diseases in three aspects: increasing the attention to mental health of older adults, improving their abilities of daily life and improving the strength of economic security.

## Data availability statement

The original contributions presented in the study are included in the article/Supplementary material, further inquiries can be directed to the corresponding author/s.

## Ethics statement

The studies involving humans were approved by the Ethics Review Committee of Peking University. The studies were conducted in accordance with the local legislation and institutional requirements. Written informed consent for participation in this study was provided by the participants’ legal guardians/next of kin.

## Author contributions

ZN: Data curation, Writing – original draft. XZ: Data curation, Writing – original draft. KT: Writing – review & editing. QC: Writing – review & editing. YY: Conceptualization, Writing – review & editing. SX: Conceptualization, Funding acquisition, Writing – review & editing.
